# Interaction of the intestinal cytokines-JAKs-STAT3 and 5 axes with RNA N6-methyladenosine to promote chronic inflammation-induced colorectal cancer

**DOI:** 10.3389/fonc.2024.1352845

**Published:** 2024-07-29

**Authors:** Nardana Esmaeili, Ahmed Bakheet, William Tse, Shujun Liu, Xiaonan Han

**Affiliations:** ^1^ Division of Hematology and Oncology, Department of Medicine, MetroHealth Medical Center (MHMC), Case Western Reserve University (CWRU) School of Medicine, Cleveland, OH, United States; ^2^ Division of Cancer Biology, Department of Medicine, MetroHealth Medical Center (MHMC), Case Western Reserve University (CWRU) School of Medicine, Cleveland, OH, United States; ^3^ Cancer Genomics and Epigenomics Program, Case Comprehensive Cancer Center, Case Western Reserve University (CWRU), Cleveland, OH, United States

**Keywords:** JAKs, STAT3, STAT5, N6-methylAdenosine (m6A), chronic inflammation, colorectal cancer

## Abstract

Colorectal cancer (CRC) is one of the most common cancers, with a high mortality rate worldwide. Mounting evidence indicates that mRNA modifications are crucial in RNA metabolism, transcription, processing, splicing, degradation, and translation. Studies show that N6-methyladenosine (m6A) is mammalians’ most common epi-transcriptomic modification. It has been demonstrated that m6A is involved in cancer formation, progression, invasion, and metastasis, suggesting it could be a potential biomarker for CRC diagnosis and developing therapeutics. Cytokines, growth factors, and hormones function in JAK/STAT3/5 signaling pathway, and they could regulate the intestinal response to infection, inflammation, and tumorigenesis. Reports show that the JAK/STAT3/5 pathway is involved in CRC development. However, the underlying mechanism is still unclear. Signal Transducer and Activator of Transcription 3/5 (STAT3, STAT5) can act as oncogenes or tumor suppressors in the context of tissue types. Also, epigenetic modifications and mutations could alter the balance between pro-oncogenic and tumor suppressor activities of the STAT3/5 signaling pathway. Thus, exploring the interaction of cytokines-JAKs-STAT3 and/or STAT5 with mRNA m6A is of great interest. This review provides a comprehensive overview of the characteristics and functions of m6A and JAKs-STAT3/5 and their relationship with gastrointestinal (GI) cancers.

## Introduction

Colorectal cancer (CRC) ranks as the third most common tumor worldwide, with high incidence and mortality rates annually. CRC is a complex disease caused by various risk factors such as environmental exposure, genetic alterations, and epigenetic modifications (1). Epigenetics is a type of genetic modification that alters gene expression with no change in the nucleotide sequence of genes. Many studies show that RNA modification is an important mechanism of epigenetic regulation, which plays a pivotal role in the occurrence of different diseases (2). RNA modification occurs on all nucleotides: A, U, C, and G (3). However, Adenine is a nucleotide that undergoes a heavy modification in RNA and poses important activities (4). It has been well established that mRNA modification, especially N6-methyladenosine (m6A), mediates various fundamental biological processes (5). The m6A methylation is a reversible process in eukaryotes carried out by methyltransferases and demethylases (6–8). Several studies suggest that m6A methylation is associated with various cancers. Emerging evidence indicates the critical role of the m6A epi-transcriptome in every characteristic of cancer biology. Among all epigenetic modifications, m6A plays a crucial role in the progression and development of CRC (9). In recent years, many studies focused on the significance of m6A in regulating gene expression and disease progression, and several genes have been identified as the new m6A methylation regulation molecules. However, their function and mechanism have not been fully understood.

On the other hand, cell fate, survival, and genome maintenance are regulated via the Janus Kinases/Signal Transducer and Activator of Transcription (JAK/STAT) pathway (10). In general, binding a ligand to a growth factor or cytokine receptor launches the JAK/STAT signaling pathway. The growth factor receptors are auto-phosphorylated in the JAK-independent pathway; however, JAK phosphorylates the tyrosine residues in the JAK-dependent pathway. These phosphorylated sites would later provide docking sites for SH2 domain-containing molecules such as STAT5A/B (11). The crystallization study confirmed the antiparallel dimerization mode of unphosphorylated STATs that switches to parallel dimers upon phosphorylation. This shift between phosphorylation and dephosphorylation modes is the most efficient nucleus translocation form (12). The JAK/STAT pathway also plays a vital role in gene expression in eukaryotic cells. It was shown that STAT3 is highly conserved across different species. For example, STAT3 isolated from the Tasmanian devil facial tumor disease shares more than 99% amino acid sequence homology with human STAT3 orthologue (11). Despite being a transcription factor, STAT5 is essential in gene expression regulation. The complex formation of STAT5 protein requires three different dimer interfaces: (1) the N-domain for oligomerization, (2) the coiled-coil domain for DNA binding, and (3) the SH2 domain, which is required for dimerization via SH2 domain-pY residue. The STAT5A and STAT5B genes are located on chromosome 17q21.2 and show around 92% identity (731 amino acids out of 794). The STAT5A and STAT5B are mainly different at the C-terminal and N-terminal ends. In addition, the DNA binding properties of STAT5A and STAT5B are also different, where STAT5B forms solid bonds with DNA with very different spacing to palindromic invert repeat sequences, which impacts gene regulation. STAT5A and STAT5B show specific functions such as different protein-protein interactions, chromatin assembly, or variation in protein turnover and expression (11). Reports indicate that STAT5B is expressed in natural killer muscle cells, liver hepatocytes, liver endothelium, and cholangiocytes, while STAT5A is mainly expressed in mammary gland epithelial cells (13). The STAT5A and STAT5B proteins are activated by cytokines and hormones such as prolactin and growth hormone (14). STAT5 is also triggered by growth factors such as stem cell factor, FLT3 ligand, epidermal growth factor-, fibroblast growth factor-, platelet-derived growth factor-family members, and other cytokines/growth factors/chemokines (13). Tyrosine phosphorylation, serine/threonine phosphorylation, and other post-translational modifications, such as acetylation/sumoylation, regulate STAT5 activity (11). A large number of studies have reviewed the signaling pathway of STAT3. However, the function of intestinal STAT5 has not been reviewed. In this review, we overview the role and the interplay between mRNA m6A and the JAKs-STAT3 and 5 axes in gastrointestinal (GI) cancer with emphasis on STAT5.

## An overview of RNA modification of m6A

It has been well established that methylation of the adenosine base at the nitrogen-6 position is the most abundant internal RNA modification (15). N6-methyladenosine (m6A) is considered one of the most common RNA methylation modifications that function in RNA processing, transport, and other functions (16). In general, m6A methylation modulates target gene expression through changing mRNA stabilization, splicing, degradation, and translation efficiency. The function of m6A has been elaborated in different biological procedures, including stem cell differentiation, embryonic development, DNA damage, and tumor progression (9, 17).

**Figure 1:**
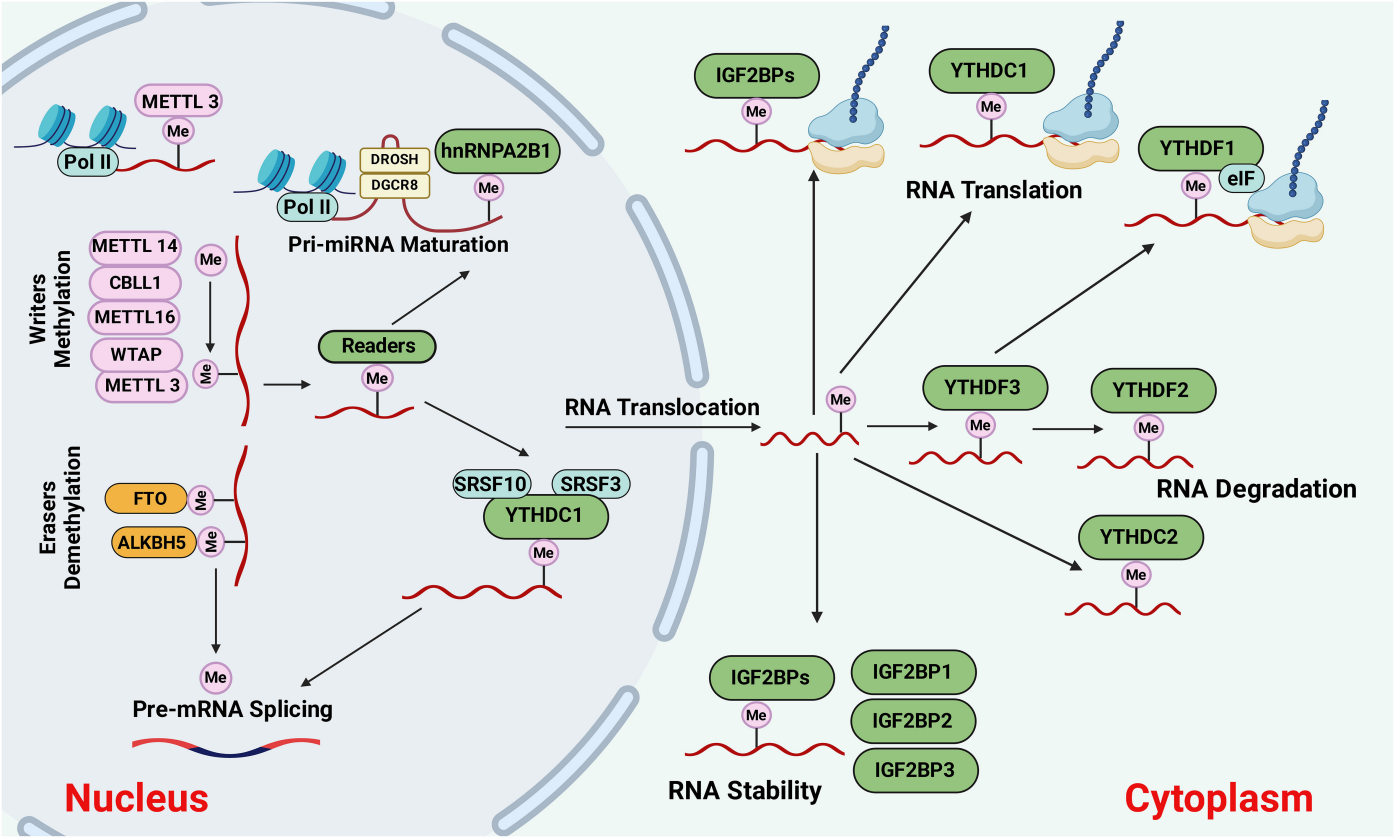
The causes and outcomes of m6A methylation. M6A methylation is catalyzed by the writers, including METTL3, METTL14, METTL16, WTAP, or CBLL1. Demethylases such as FTO and ALKBH5 erase the m6A modification through demethylation. The m6A-modified RNAs are recognized by reader proteins, including YTHDC1/2, IGFBP1/2/3, YTHDF1,2,3, and exported to the cytoplasm for degradation, protein translation, and so on. Figure created with BioRender.

The m6A methylation is determined by a methyltransferase (writers), demethylase (erasers), and binding proteins (readers) (18). The overall picture of m6A methylation is presented in **Figure 1**. In addition, m6A is abundant in mRNA and non-coding RNA (ncRNA), and its function in ncRNA metabolism is essential (19). To date, 11 readers, seven writers, and two erasers have been identified. In the following sections, we will cover these three components of m6A modification.

### m6A writers

Writers carry the beginning process of m6A methylation; different methyltransferases could form a complex to gain more robust catalytic ability. The methyltransferase complex (MTC) contains the m6A/METTL complex (MAC) and the m6A/METTL-associated complex (MACOM) (20). On the other hand, MAC has METTL3 and METTL14, which can form a stable heterodimer, which plays a vital role during m6A deposition on nuclear RNAs. METTL14 interacts with METTL3 through their methyltransferase domains (MTD), where METTL3 serves as the methyl catalytic core, while METTL14 stabilizes METTL3 to exert methyltransferase activity and provides an RNA-binding platform (6). However, reports show that METTL3 can function independently from METTL14 and promote the translation of specific mRNAs (21). There is around 22% shared sequence identity between METTL14 and METTL3 (22).

Different types of “writers” (e.g., METTL 3/14/16, WTAP, KIAA 1429, RBM 15/15B, and ZC3H13) are involved in catalyzing the m6A methylation on the mRNA (16). Another type of writer is Wilms’ tumor 1-associating protein (WTAP), which forms a complex with METTL3 and METTL14 that regulates m6A. WTAP is universally expressed in the nucleus and acts as a guide to recruit the methyltransferase complex to target transcripts (23). Although the loss of METTL14 promoted tumor proliferation *in vivo*, no impacts on the proliferation of CRC cells *in vitro* were observed. Moreover, the deletion of METTL14 prevents the embryonic stem cell potential for self-renewal and differentiation (24). A recent study by Wang et al. (25) explained the METTL14 role in suppressing CRC metastasis. They also reported that METTL14 is decreased in CRC tissues and correlated with CRC patients’ prognosis. In addition, suppression of METTL14 increased the mRNA stability of the arrestin domain containing 4 (ARRDC4), a downstream m6A target of METTL14, via an m6A-YTHDF2-dependent pathway (25).

Studies show that m6A regulates noncoding RNA (ncRNA) expression through a “writer” complex (9, 26). For instance, METTL3 is upregulated in metastasis to promote cell migration and invasion in CRCs by altering miR-1246 expression, while METTL14 suppresses cell proliferation, invasion, and migration in CRCs via miR-375 (27). METTL3 is also involved in tumor progression of other cancers such as leukemia and bladder and gastric cancers via regulation of their downstream genes (17). Studies on methyltransferases in kidney cancer, such as ccRCC, demonstrate that both METTL3 and METTL14 play as tumor suppressors (28–30). METTL3 was shown to be involved in the tumorigenesis and metastasis of colorectal cancer trough YPEL5 expression inhibition in a YTHDF2-dependent manner (31). Most studies on methyltransferases in bladder cancer claim that METTL3 and METLL14 are oncogenes and tumor suppressors, respectively (32).

### m6A erasers

Several m6A-specific erasers, AlkB homolog 5 (ALKBH5), and fat mass-and obesity-associated protein (FTO) have been discovered. FTO was first discovered as a gene involved in obesity and energy metabolism and later introduced as the RNA m6A demethylase (33). It is well known that “erasers” such as FTO, ALKBH 3, and ALKBH 5, demethylate m6A, while the functions of “readers” are to identify m6A and selectively bind to target transcripts (34). The discovery of FTO suggested that RNA modification is reversible and dynamic (15). FTO carries out the m6A demethylation process in a Fe(II)- and α-ketoglutarate-dependent enzymatic reaction (35). Functionally, FTO generally acts as an oncogene in different cancers, such as glioblastoma and melanoma, emphasizing the potential of targeting FTO as a therapeutic approach against cancer (36). However, there are several inconsistencies regarding the functions of the FTO in tumor development and prognosis. For example, several reports indicate that FTO has tumor suppressor activities on CRC invasiveness and metastasis (33), ovarian cancer stem cell self-renewal (37), and papillary thyroid cancer (38). Also, a high relapse rate and poor prognosis in CRC patients were attributed to hypoxia-induced downregulation of FTO protein levels but not RNA. This hypoxia-induced FTO depletion results from ubiquitin-mediated protein proteasome-associated degradation (33).

### m6A-readers

m6A readers such as YTHDC1/2, YTHDF1/2/3, insulin-like growth factor 2 mRNA-binding proteins (IGF2BP1/2/3), HNRNP, and eIF3 can recognize the m6A residues. YTH domain family protein 1 (YTHDF1) is an example of an m6A’ reader’ that enhances the translation efficacy of m6A-modified mRNAs and plays as an oncogene in human cancers. It was shown that high YTHDF1 expression is associated with poor prognosis in hepatocellular carcinoma (HCC) and CRCS. A recent study by Wang et al. (39) found that YTHDF1 is highly upregulated in CRC, supporting the concept that YTHDF1 may convert the deregulated m6A modifications to pro-tumorigenic signals. The expression level of YTHDF1 in human CRC is positively correlated with metastatic progression. Findings from the Ythdf1-knockout mice, CRC cell lines, and primary CRC organoids demonstrated that YTHDF1 executes its pro-tumorigenic impacts by enhancing tumor growth, migration, invasion, and metastasis (39).

In CRC, the epithelial-to-mesenchymal transition (EMT) process, in which cells lose their epithelial traits and acquire mesenchymal features, correlates with a more invasive or metastatic behavior. Throughout the process of EMT, tumor cells experience the breakdown of tight junctions, disturbance in apical-basal polarity, and restructuring of their cytoskeletal framework, facilitating the development of an invasive nature. EMT is irregularly governed by external stimuli originating from the tumor microenvironment within cancer cells, encompassing growth factors and inflammatory cytokines alongside internal physical pressures like hypoxia (40). It was reported that angiogenesis levels are elevated in the early stages of CRC growth. However, this process doesn’t show a consistent increase but exhibits an oscillatory pattern (41). EMT regulation demands a robust transcriptional apparatus primarily composed of developmental transcription factors. These factors orchestrate the modulation of epithelial and mesenchymal markers in a synchronized manner. The main groups of EMT-activating transcription factors include the SNAIL family (SNAIL/SLUG), the zinc finger E-box binding homeobox (ZEB) family (ZEB1/ZEB2), and the TWIST family of basic helix-loop-helix (bHLH) transcription factors (TWIST1/TWIST2) (40). Lin et al. (42) presented the importance of m6A modification on EMT regulation in cancer cells and the translation of Snail, an EMT key transcription factor, during this process. They showed that m6A modification levels in mRNA were significantly increased in cancer cells undergoing EMT compared to normal cells. In addition, there is a higher binding affinity between YTHDF1 and CDS of Snail mRNA in cancer cells undergoing EMT. In addition, the deletion of METTL3 and overexpression of ALKBH5 resulted in suppression of the *in vitro* migration, invasion, and EMT of cancer cells. The loss and gain functional studies also demonstrated that YTHDF1 mediates m6A-increased translation of Snail mRNA (42). In addition, the proliferation and growth of HCC cells are inhibited by YTHDF2 through disruption of the stability of epidermal growth factor receptor mRNA. YTHDF3 also negatively modulates the interaction between two long noncoding RNAs, growth arrest-specific 5 (GAS5) and yes-associated protein (YAP), leading to the inhibition of CRC progression (43). It has been well established that IGF2BPs can improve mRNA stability by binding to target transcripts through an m6A motif64 of GG(m6A)C (44). Studies revealed that “readers” such as YTHDF1, IGF2BP1, IGF2BP3, and EIF3B that identify m6A modulation sites are also regulated by ncRNAs (45).

The accumulating evidence revealed that noncoding RNAs (ncRNAs), including microRNA (miRNA), circular RNA (circRNA), and long noncoding RNA (lncRNAs), are involved in the initiation and development of colorectal cancer (1). Circular RNAs are a group of non-coding RNAs without 5′ caps and 3′ polyadenylated tails involved in CRC (46). In addition, the dysregulation of circRNAs leads to the chemoresistance of CRC. Emerging reports suggest that circRNA/microRNA (miRNA)/mRNA regulatory networks play an important role in CRC development and treatment (47). For instance, circ_0007142 increased cell proliferation and metastasis in CRC by regulating the miR-455–5p/SGK1 axis (48). Furthermore, miRNAs and lncRNAs play a pivotal role in diagnosing, prognosis, and treating CRC (49). The miRNAs can function as oncogenes or tumor suppressors, depending on their altered pathways and primary location, such as colon or rectal cancer (50). For instance, miRNA-9 and miRNA-101 act as tumor suppressors in CRC by suppressing colon cancer cell migration (50, 51). On the other hand, miRNA-200, miRNA-17, and miR-141 are three examples of CRC oncogenes that inhibit different tumor suppressor genes and promote cancer cell proliferation (52–54).

It was shown that the higher concentrations of circRNAs positively increase the m6A levels (55). Studies revealed that the sites of circRNAs modified by m6A differ from those of mRNAs modified by m6A, indicating that disruption in the m6A modification of mRNA does not correspond to the m6A circRNAs distraction. Thus, to eliminate m6A modification effects, it is also required to consider the m6A of circRNAs (27). The m6A RNA modifications are dynamic and reversible and regulate RNA metabolism, which can alter the genetic information at the mRNA level. The m6A RNA modifications can also modulate the balanced mRNA expression and facilitate mRNA translation, thereby affecting the levels of the target genes involved in proteins and RNA metabolism. It was shown that RNA metabolism impacts the occurrence and deterioration of diseases, such as cancer and inflammation (56).

In the context of the intestine, m6A is involved in various processes, including intestinal stem cell maintenance and differentiation, intestinal epithelial cells proliferation and regeneration, intestinal epithelial barrier function, and host-microbe interactions. For example, m6A modification of the RNA molecule has been shown to regulate the expression of critical genes involved in intestinal stem cell maintenance and differentiation, which are crucial for the regeneration and repair of the intestinal epithelium. Also, m6A has been implicated in regulating host-microbe interactions in the gut, which plays a critical role in maintaining gut homeostasis and preventing the development of inflammatory bowel diseases. Overall, m6A modification is a crucial process that plays a key role in various biological processes in the intestine and holds significant promise for developing new therapeutic strategies for treating gut-related diseases.

## The function of m6A in colorectal cancer

Around 2 million Europeans and more than 1.5 million North Americans suffer from inflammatory bowel disease (57). Colorectal cancer (CRC) is the second leading cause of cancer-related death worldwide, and metastasis is considered the primary cause of cancer death (58). Many epidemiological data link inflammation to cancer within the digestive system. Inflammation is caused by poor diet, gut microbiota, and widespread infection. For instance, Helicobacter pylori infection causes inflammation in the gut, leading to around 75% of gastric cancers (59). Studies show that the risk of developing colon cancer due to chronic inflammation is very high in autoimmune disorders, ulcerative colitis, and Crohn’s disease (60). Data shows that around 50% to 60% of CRC patients ultimately develop metastatic disease, mainly affecting the liver and lungs (39). Despite recent advances in CRC treatments, the survival rate for patients with postoperative and advanced CRC recurrence remains low. Since CRC is mainly diagnosed at an advanced stage of metastasis, the death rate related to CRC metastasis is high (61). Moreover, CRC is highly heterogeneous, making it a very complex disease. Therefore, there is an urgent need to develop sensitive biomarkers capable of accurate prognosis prediction and monitoring therapeutic effects in CRC patients (62). Since epigenetic changes impact tumorigenesis and the progression of CRC, they can be used as potential clinical biomarkers for prognostic and therapeutic uses of CRC (63).

Post-transcriptional modifications of the RNA transcriptome, known as epi-transcriptomics, play critical regulatory roles in gene expression. Recent advances in RNA sequencing technologies discovered various RNA modifications on a transcriptome-wide scale, suggesting that dysregulation of RNA modifications results in tumorigenesis (39). It is well established that CRC occurrence and development are associated with the changes in levels of m6A RNA methylation and m6A RNA methylation regulators (57). Studies show that the amounts of m6A RNA methylation and the expression of its regulatory factors can impact cell proliferation, occurrence, metastasis, stemness-like properties of cancer cells, and invasion in colon cancer (CC) (19, 59). However, the function of m6A RNA modifications in rectal cancer (RC) is not fully understood (59). Recently, methylated RNA immunoprecipitation sequencing (MeRIP-seq) analysis of CRC patients demonstrated that m6A peaks are present in most mRNAs where m6A peaks are differentially methylated (60).

M6A methylation is the most common modification in eukaryote mRNA that functions as both an oncogene and a tumor suppressor in cancer metastasis and the EMT process (64). m6A plays a critical role in various cancers, including leukemia, brain, cervical, endometrial, breast, liver, and lung cancers (32). A regulatory function of m6A has been shown in oncogenesis and development by modifying different target genes (65, 66). Mutation of oncogenes and tumor suppressor genes caused by m6A regulatory factors can affect cancer cell proliferation, metastasis, and infiltration (58). The methylation of m6A regulates miRNA synthesis, processing, and maturation, which are crucial in tumorigenesis and cancer progression (62). The m6A modification also changes the structure of local RNA at the terminal loop region of primary miRNAs (pri-miRNAs), thus stimulating their processing through nuclear transcripts and alternative splicing by modulating RALY binding. A recent study by Wang et al. (63) unraveled an antiviral function for m6A modification in the small intestine during rotavirus infection through ALKBH5. The depletion of Mettl3 in IECs of mice improved their resistance to RV infection and increased the expression of interferons (IFNs) and IFN-stimulated genes (ISGs) (63).

The abnormal methylation of m6A mRNA in CRC could benefit CRC prognosis. Many studies show that RNA m6A regulatory factors, such as METTL3, METTL14, WTAP, FTO, YTHDC1, and YTHDF3, are abnormally expressed in CRC. METTL3 is a 70 kDa protein first identified in Hela cell lysates (67). METTL3 mediates the m6A methylation on mammalian RNAs and is crucial in influencing angiogenesis and promoting tumor progression (68). In CRC tissues, the METTL3 expression is significantly higher than in normal tissues, indicating that METTL3 plays a pivotal role in CRC (57, 69). A recent study (70) revealed that METTL3 upregulation in CRC tissues results in low survival in CRC metastasis. It also improved the stability of PLAU mRNA and promoted CRC cell metastasis through m6A modification. These findings provide novel therapeutic targets for treating CRC metastasis (70).

On the other hand, METTL3 has also been demonstrated as a tumor suppressor in CRC. A recent study (71) claimed that a high content of METTL3 in CRC patients is not beneficial for the cancer cells’ growth and division, and it also suppresses CRC cell proliferation, migration, and invasion through p38/ERK pathways, suggesting that METTL3 can be considered that a prognostic factor in CRC patients. In addition, regulators of m6A RNA modification can prevent the occurrence and development of CRC by altering its protein expression level and the protein expression levels of its downstream targets in CRC (57). M6A methylation in lncRNA is also required for cancer cell proliferation, metastasis, and stemness-like properties, including colorectal cancer (CRC) (19). Recently, Zhang et al. (72) also showed that there is a positive correlation between METTL3, LINC00662 (a lncRNA with a length of 2097 nt), and vascular endothelial growth factor A (VEGFA) in CRC tissues. In addition, it was demonstrated that METTL3 dually modulates the stability of the LINC00662 and VEGFA RNAs, thus promoting angiogenesis in CRC. These results together indicate that METTL3 increases CRC progression (72). The expression level of long intergenic noncoding RNA 460 (LINC00460) was significantly increased in CRC and regulated its growth and metastasis *in vitro* and *in vivo* (73). Also, LINC00460 could promote mRNA stability of HMGA1 via interacting with IGF2BP2 and (DHX9), which leads to a biological response to CRC malignant proliferation and metastasis. Furthermore, m6A modification of HMGA1 mRNA decreased its expression in CRC, and HMGA1 expression regulated by LINC00460 is METTL3-dependant (73).

In tumor tissues, ZEB1-AS1 was significantly overexpressed, which was related to the metastasis of EMT, indicating that ZEB1-AS1 level could be a valuable indicator for predicting the progression and prognosis of CRC (62). METTL3 also enables tumor progression by upregulating lncRNA RP11 and ZEB1 (74) or via the maturation of pre-miR-1246 (75). The METTL3 and METTL14 writers were shown to suppress CRC proliferation and migration via the p38/ERK pathway (71). Methyl CpG binding protein 2 (MeCP2) is a methylated DNA binding protein. Its oncogenic functions in gastric and colorectal cancer and facilitating metastasis of CRC have been documented. In addition, the interaction between MeCP2 and METTL14 was shown to modulate m6A methylation in CRC. In addition, the CRC tumor samples showed a higher expression level of MeCP2, indicating that MeCP2 might act as an oncogene in CRC (6). The m6A methylation regulation function of various studies. However, new reports claim that METTL14 impacts downstream events more than METTL3. Also, the downregulation of METTL14 and YTHDC2 is associated with the poor prognosis of rectal cancer patients (59). METTL14 downregulation in rectal cancer results in reduced immune cell infiltration and poor prognosis indicating that METTL14 expression level could be utilized as an independent prognostic factor in rectal cancer (76).

It was shown that m6A-modified mRNAs are recognized by IGF2BPs, leading to enhanced target mRNA stability, such as MYC, in an m6A-dependent manner (77). IGF2BPs also contain oncogenic roles in cancer cells by stabilizing methylated mRNAs of oncogenic (77). Moreover, Erasers can cause the progression and migration of CRC cells. For example, FTO is involved in the degradation of miR-1266 or reducing expression levels of STAT3, cyclin D, and MMPs to stimulate tumor growth (78). Recently, Bai et al. (79) demonstrated that the CRC cell’s tumorigenicity *in vitro* was dramatically suppressed when the expression of YTHDF1 was knocked down. In addition, YTHDF1 silencing inhibited the colonosphere formation ability *in vitro* and Wnt/β-catenin pathway activity in CRC cells. This compelling evidence suggests that YTHDF1 is overexpressed in CRC and functions as an oncogene in CRC (79). According to Zhang et al. (80), the lncRNA NEAT1 is demethylated by ALKBH5, which results in gastric cancer invasion and metastasis through altering the expression of EZH2. On the other hand, ALKBH5 was demonstrated to suppress gastric cancer invasion by downregulating and removing the m6A modifications of PKMYT1 (81). ALKBH5 is an oncogene that accelerates gastric cancer proliferation, metastasis, and invasion (82).

CircRNAs are classified as noncoding RNAs (ncRNAs) and characterized by the covalently closed loop structure without a 3′-poly-A tail or 5′-cap. In general, circRNAs are more stable and resistant to RNA exonuclease degradation. They are valuable prognostic biomarkers and promising targets for treating human cancers (83). YTHDC1 is one of the m6A readers that play critical roles in different cancers, and a high concentration of YTHDC1 was reported in CRC cells and tissues (84). Recently, a direct interaction between YTHDC1 and circFNDC3B in CRC cells was demonstrated. In addition, cytoplasmic export of circFNDC3B in LoVo and HCT116 cells requires YTHDC1, indicating the significance of m6A modification in circ-RNAs (85). A recent study showed that circ_0003215 is downregulated in CRC, and the functional assays demonstrated that the malignancy of CRC was inhibited in both *in vitro* and *in vivo*. Furthermore, circ_0003215 also has m6A methylation, which results in RNA degradation by m6A reader protein YTHDF2 (86). A newly identified circRNA, circ1662, is derived from 3 neighboring exons in the yes-associated protein 1 (YAP1) gene, which seems to play an oncogenic role in CRC, promoting CRC cell invasion and migration. It was reported that circ1662 formation is regulated by METTL3-initiated m6A methylation in CRC cells, and METTL3 apparently can accelerate CRC metastasis using the regulatory mechanism of circ1662. Since circ1662 is positively associated with METTL3 and YAP1 protein expression, it was suggested that circ1662 could be employed as a biomarker to identify cancer metastasis (87). The critical function of m6A-related lncRNAs in the tumor microenvironment (TME) remodeling has been demonstrated (62). Upon cancer development, IL-6 and IL-8 are secreted by tumor-infiltrating immune cells within the tumor microenvironment (TME) (88). A recent study by Liu et al. (89) showed that MIR100HG utilizes a miRNA-independent role in EMT regulation and metastasis in CRC cells by forming a regulatory circuit involving hnRNPA2B1 and TCF7L2. Data collected from 473 CRC specimens and 41 para-cancer tissues established a powerful prognostic model based on 16 genes out of 37 m6A-modified prognostic lncRNAs (90).

The involvement of the epithelial growth factor receptor (EGFR) signaling pathway in CRC progression makes EGFR a valuable therapeutic target in developing tumor-targeted therapeutic drugs. The fragile X mental retardation 1 gene, *FMR1*, is located on human chromosome Xq27.3, which encodes the FMR1 protein, an RNA-binding protein (RBP). It was shown that the FMR1 protein plays a vital role in the growth and progression of various tumors (91). Although the function of FMR1 in regulating CRC tumorigenesis and EGFR signaling pathway is not fully understood, a recent study (92) demonstrated that FMR1 is upregulated in CRC, which is associated with the proliferation and migration of CRC cells. Moreover, FMR1 was shown to recognize the m6A-modification site in EGFR and retained its expression in an m6A-dependent manner. In addition, the FMR1 knockdown effects in CRC cells were eliminated by METTL3, indicating that the METTL3/FMR1/EGFR complex is involved in CRC progression (92).

Mutation in m6A sites of RNA could affect m6A deposition and causes abnormal post-transcriptional regulation, which might result in carcinogenesis (93). The missense rs8100241 variant found in the exon of Ankyrin Repeat and LEM Domain Containing1 (ANKLE1) with a G>A change is linked to reduced CRC risk. Although variant ANKLE1 [A] is methylated by METTL3, ANKLE [G] could not be methylated. This phenomenon improves the ANKLE1 mRNA stability via m6A, thus decreasing CRC risk (94). Overall, epigenetic changes play a pivotal role in the epithelial-to-mesenchymal transition, a crucial mechanism for metastasis, and mainly include valuable biomarkers in CRCs such as DNA methylation, ncRNA m6A, and mRNA m6A. RNA m6A modification is critical for colorectal organ homeostasis, and its disruption leads to inflammatory disorders and aggressive cancers (19).

## m6A modification and its potential in targeted therapy for m6A

Targeted therapy focused on m6A modification has emerged as a prominent area of research for developing new drugs in recent years. It was shown that m6A modifications play an important function in cancer responses to chemotherapy, radiotherapy and immunotherapy, suggesting that m6A regulators could be targeted and used in combination with chemotherapy, radiotherapy, or immunotherapy to treat cancer. The primary targets for m6A modification therapy include FTO inhibitors, METTL3–14 activators/inhibitors that could be used in combination with chemotherapy and immunotherapy (95). The role of METTL3 in CRC cancer has been investigated. Recently, Chen et al. (69) showed that increased expression of METTL3 results in a poor prognosis of CRC while Mettl3 knockout reduces colorectal tumorigenesis. The study also elaborated that the GLUT1-mTORC1 axis is the main METTL3 target in CRC and targeting METTL3 and mTORC1 has a significant potential to inhibit CRC growth, suggesting that METTL3 could be used as a target to treat patients with CRC (69). The association between METTL3 expression and the disease control rate in colorectal cancer (CRC) patients was explored by Li et al. (96). The findings of this study revealed that patients with elevated METTL3 expression had a lower response to chemotherapy leading to poorer treatment outcomes. METTL3 also promotes CRC stemness which consequently contributes to the development of resistance to chemotherapy in CRC patients. However, the knockdown of METTL3 in SW620 and HCT116 cells resulted in higher sensitivity to oxaliplatin-based chemotherapy, compared to the control. In addition, PDX tumor models were injected with METTL3 siRNA to assess the therapeutic effect of METTL3. The IHC results of isolated tumors demonstrated that METTL3 expression significantly reduced in the experimental group treated with METTL3 siRNA. These findings suggest a promising therapeutic scheme for CRC via application of METTL3 inhibitors (96).

The aerobic glycolysis pathway in tumor cells known as the Warburg effect is an abnormal glycolysis that enhances glucose uptake, ATP, and lactate production, promoting tumorigenesis. A recent study by Yang et al. (97) focused on illustrating the role of m6A modification in the Warburg effect in CRC. The study showed that METTL3 knockdown represses Warburg effect in CRC via regulating HIF-1α suggesting that METTL3 is a potential diagnostic marker and therapeutic target (97). Moreover, inhibition of m6A modification due to METTL3 and METTL14 deletion facilitates the IFN-γ-Stat1-Irf1 signal transduction through YTHDF2, resulting in the stabilization of STAT1 and Irf1 mRNA. Consequently, the response to anti-PD-1 therapy is enhanced, highlighting the potential of METTL3 and METTL14 as therapeutic targets for anti-cancer immunotherapy (98).

Recently You et al. (99) constructed small extracellular vesicles (sEVs) that highly express CD47 and increased cyclic arginine–glycine–aspartic modification. This novel strategy was shown to effectively deliver siYTHDF1 to treat gastric cancer with less toxicity via depletion of YTHDF1 leading to suppression of GI cancer progression and metastasis (99). FTO is an essential m6A regulator which its overexpression increases proliferation and migratory potential in MKN28 cells. However, the FTO knockdown suppresses the tumor growth in HGC27 xenograft model. In addition FTO changes the expression pattern of EMT-related genes including E-cadherin and vimentin indicating that FTO possibly functions in the EMT pathway as an oncogene (100). Chemotherapy drug resistance is a major hindrance to achieving treatment in cancers. A potential therapeutic approach to mitigate chemoresistance involves targeting m6A RNA modification. It was shown that YTHDF1 suppression enhances the effectiveness of 5-Fluorouracil and cisplatin chemotherapy drugs in drug-resistant CRC cells (101, 102). Mounting evidence shows that m6A regulation has the potential to be used in targeted therapy for IBD and CRC diseases. There is an urgent need to investigate novel therapeutic strategies targeting m6A for translational clinical applications in IBD and colorectal cancer.

## Bioinformatics analysis to study the correlation between m6A RNA methylation and colorectal cancer

In recent years, bioinformatics has emerged as a powerful tool in elucidating the molecular complexities of cancer biology. Combining the capabilities of bioinformatics with experimental evidence enables researchers to explore the interplay between m6A modification and CRC pathogenesis, shedding light on novel biomarkers, therapeutic targets, and potential prognostic indicators.

m6A2Target was the pioneering resource developed in 2020 for targets of m6A writers, erasers, and readers (103). Recently, an updated database called RM2Target has been published (http://rm2target.canceromics.org/). It is more powerful than m6A2Target, encompasses a broader range of RNA modifications, and compiles a significantly larger number of target gene associations (104). The M6ADD database is another platform developed to explore the association between m6A modification and gene disorders and diseases, which consists of 222 m6A-related diseases from both humans and mice. The development of the m6ADD database aims to facilitate researchers in acquiring insights into the functions of particular genes and specific gene-protein interactions (105). The RMDisease, is another example of an RNA modification database developed to address the genetic variants affecting RNA modifications and their potential association with diseases. This resource integrates data from a large number of RNA modification sites and somatic and germline SNPs, serving as a useful mapping resource for exploring different genetic factors involved in epitranscriptome regulatory pathways and their role in diseases (106). A recently upgraded version of RMDisease database, RMDisease V2.0, has become available by compiling all existing RNA modifications-associated variants and annotating their potential implications in diseases and traits, enabling it to cover a lager range of RNA modification types in different species (107). The m6A-Atlas was released in 2021, offering a more comprehensive perspective of the m6A epitranscriptome. This database resource integrated different m6A sites from seven high-resolution epitranscriptome profiling and diverse post-transcriptional regulatory mechanisms (108). Recently, the m6A-Atlas v2.0 became available to users, enabling them to filter next-generation sequencing results. This database provides a free resource for different m6A enrichment regions for users to screen and filter data.

Furthermore, by integrating annotation data, m6A-Atlas v2.0 facilitates exploring relationships between RNA m6A modification sites and different downstream functional characteristics (109). Human RNA Modifications Disease Database (HRMDD) is another web resource developed recently by collecting 2082 experimentally supported RNA modification-disease associations. The Cancer Genome Atlas (TCGA) analysis was used to evaluate the molecular and clinical aspects of RNA modification regulators in 33 different cancer types, and the roles of RNA modification regulator genes in cancers were visualized and characterized by the development of a regulator-Tool (110). Given that numerous studies evaluating the function of m6A regulators in CRC have been published, further in-depth study into CRC is still required to enhance patient prognosis. Therefore, bioinformatics analysis could be used as an interdisciplinary approach to improve our understanding of CRC at the molecular level by aiding researchers in efficiently and promptly identifying candidate genes. It holds promise for personalized medicine and precision oncology interventions.

## An overview of JAKs/STAT signaling pathway

Inflammation is an essential sign of carcinogenesis and tumor progression. It was shown that chronic inflammation is the cause of around 15–25% of cancer cases or deaths worldwide (111). For instance, inflammation resulting from severe *Helicobacter pylori* infection is associated with approximately 75% of gastric cancers. In addition, chronic inflammation resulting from autoimmune disorders, ulcerative colitis, and Crohn’s disease enhances the risk of developing colon cancer (112). High levels of cytokines usually characterize inflammation, including interleukin (IL)-6, IL-8, interferon (IFN)γ, tumor growth factor (TGF)-β, tumor necrosis factor-α, vascular–endothelial growth factor and nitric oxide (NO). Reports show that the upregulation of these factors in response to inflammation causes oxidative stress, resulting in DNA damage (111).

**Figure 2:**
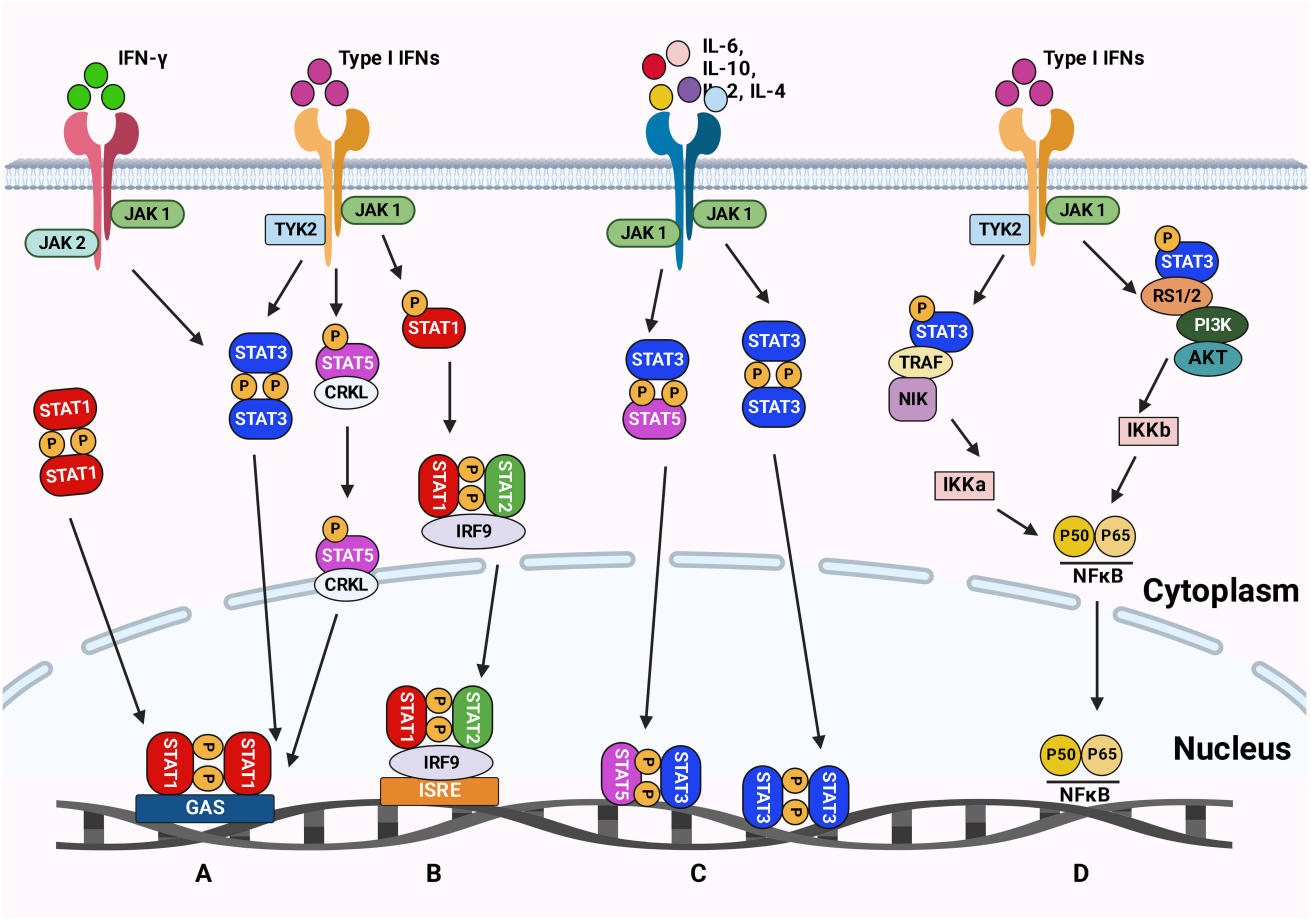
The JAK-STAT signaling pathways. **(A)** The type II IFN signaling pathway leads to phosphorylation of STAT1 and induction of inflammatory response. **(B)** The type I IFN signaling pathway results in the phosphorylation of STAT1 and STAT2, which causes an antiviral response. **(C)** Cytokines signal transduction is mediated by JAKs complexes leading to STAT3 and STAT5 phosphorylation. These phosphorylated STATs are then translocated to the nucleus to trigger the transcription of genes involved in inflammation, angiogenesis, and survival. **(D)** The Type I IFN signaling via TYK2 and JAK1 activates the NFκB pathway resulting in a viral response. Figure created with BioRender.

The JAK/STAT pathway was first discovered in 1990 when Fu et al. (113) found that the transcriptional activator interferon-stimulated gene factor 3 (ISGF3) responds to IFN-α and is comprised of multiple interacting polypeptide chains (**Figure 2**). Later on, a proposed model for the signal transduction pathway, which IFN-α induces, demonstrated the signal transduction mode of the JAK/STAT signaling pathway (114). The JAKs/STAT pathway is a pro-tumorigenic signaling pivot that maintains the pro-inflammatory environment. As an evolutionarily conserved pathway, JAK/STAT is essential for proper cellular function. JAKs/STAT was shown to be involved in oncogenesis and progression processes, including cell proliferation, differentiation, invasion, and metastasis. Cancer-related inflammation and mutation of JAKs/STAT components result in various diseases. Many reports emphasized the importance of the JAKs/STAT pathway in malignancies and autoimmune diseases, suggesting that inhibition of the JAKs/STAT pathway could open new promising avenues to treat different diseases (115).

STATs are a family of transcription factors that regulate numerous tumor-associated genes and act as critical cellular mediators in response to various cytokines and growth factors (116). The family of STAT proteins is categorized as conserved transcription factors containing seven members (STAT1, 2, 3, 4, 5a, 5b, and 6). However, the JAK family is comprised of four members: JAK1, JAK2, JAK3, and TYK2 (115). Although differences in the STAT proteins’ structure, expression levels, and subcellular localization usually lead to variations in their cellular response, the general knowledge from one member could be applied to others since they share similar structural arrangements of their functional motifs. Since different STATs can interact with the same DNA regulatory element (DRE), the same stimulus can trigger different types of STAT. JAK-STATs pathways are involved in several biological processes. Thus, precise homeostatic mechanisms at various levels must be performed to maintain the signaling pathways.

The alteration in signal transduction functions of different JAK-STATs is carried out through auxiliary STATs recruitment, STAT competition, epigenetic modifications, and recruitment of proteins that inhibit JAK-STAT phosphorylation and DRE binding (117). STAT proteins remain in the cytoplasm when they are inactive. The cytokine receptors are phosphorylated by forming noncovalent binding with JAKs, consequently recruiting STAT proteins. In general, phosphorylation on a C-terminal tyrosine residue activates STAT protein. Thereafter activation, STAT dissociates from JAK and immediately undergoes a stable dimerization upon tyrosine-phosphorylation. They are translocated into the nucleus to modulate the expression of several target genes after binding to specific palindromic DNA elements (e.g., IFNγ-stimulated response element sites) (111, 115). Since STAT1, STAT2, and STAT3 are more stable, it is proposed that the active transcriptional region can regulate the stability of the protein. On the other hand, STAT4, STAT5, and STAT6 can be employed as targets for ubiquitin-dependent destruction (115).

In general, the loss of function or gain of function mutations in JAK-STAT is the leading cause of the initiation and development of tumorigenesis. It was shown that STAT3 and STAT5 are involved in tumor initiation and progression, while STAT1 and STAT2 play an essential role in anti-tumor defense and long-term immune response (117). For instance, a higher level of nuclear STAT5 is associated with early recurrence and decreased survival rate in prostate cancer, while STAT3 overexpression results in recurrence and poor survival in melanoma, cervical cancer, and colorectal cancer (118). Studies revealed that tumor growth rate and IFN-γ-driven tumor cell killing by NK and T cells were accelerated in STAT1 knockout mice, suggesting that loss of STAT1 negatively affects both innate and adaptive anti-tumor responses (119).

## The role of STAT3/5 in GI inflammation and CRC

The transcriptional activators of STAT5 include STAT5A and STAT5B, with 91% similarity at the amino acid level. Although STAT5A has a lower DNA-binding capacity than STAT5B, it can form tetramers and dimers while binding to DNA. However, STAT5B only forms dimer structures (115). According to Lin et al. (2012) normal function of natural killer (NK) cells development and maintenance of CD8^+^ T cell and CD4^+^CD25^+^ T cell critically depend on STAT5 tetramers (120). Different types of cytokines such as IL-3, prolactin, and the IL-2 cytokine family (e.g., IL-2, IL-4, IL-7, IL-9, and IL-15) can activate STAT5. Also, STAT5 can be activated by EGF, EPO, GM-CSF, TPO, GH, and platelet-derived growth factors (121).

In general, JAK/STAT pathway is a highly conserved signal transduction pathway that regulates different cellular mechanisms related to various diseases, including GI cancer. JAK/STAT5 has been shown to modulate intestinal mucosal immunity (122, 123). The function of intestinal JAK/STAT5 has been documented in different gut cytokines, hormones, and growth factors-mediated mucosal destruction or protection (124). It was reported that JAKs/STAT3 mediates signals from cytokines (e.g., IL-6) or growth factors (e.g., TGF-α) to the nucleus. In addition, regulating intestinal epithelial barrier integrity and transcription induction of antimicrobial peptides by IL-17 would protect the tissue from microbiota translocation and inflammation. However, other cytokines such as IL-4 and IL-13 stimulate the Tuft cell maturation, resulting in the response of parasite antigens (125).

Furthermore, IL-17 regulates intestinal epithelial barrier integrity. The signaling pathway of JAKs/STAT3 is activated upon binding cytokine ligands or growth factors to their receptors on the cell surface, resulting in JAKs activation. The activated JAKs consequently induce the phosphorylation and dimerization of STAT3, stimulating the transcription of several downstream genes (61). Reports show that the overactivation of the JAKs/STAT3 pathway is correlated with CRC-related phenotypes. On the other hand, inhibition of the JAKs/STAT3 signaling pathway induced apoptosis in CRC cells leading to tumor cell invasion and tumor growth restrain (126). The activation of the JAKs/STAT3 signaling pathway results in enhanced expression of malignant phenotypes-associated molecules, such as matrix metalloproteinases, VEGFA, bFGF, and HGF, consequently developing malignant tumor behaviors, including EMT, migration, invasion, angiogenesis, and metastasis (61). The analysis of target genes and cellular signaling pathways, including JAKs/STAT3 associated with CRC progression and metastasis, can elucidate the underlying mechanism of CRC progression and pharmacotherapy (127).

It was shown that the constitutive activation of STAT3 leads to the development of head and neck tumors, breast cancer, non-small-cell lung cancer, colorectal cancer, and hematological tumors. In addition, high expression of STAT3 and IL-6 can reduce chemotherapy sensitivity in high-grade breast cancer (128). Recently (129) showed that colorectal cancer-associated fibroblasts (CAFs) promote metastasis by upregulating leucine-rich alpha-2-glycoprotein 1 (LRG1). In addition, CAFs-secreted IL-6 (interleukin-6) is responsible for LRG1 up-regulation in CRC, which occurs through direct transactivation by STAT3 following JAK2 activation. Receptor tyrosine kinases such as epidermal growth factor receptors (EGFR) are the upstream activators of the JAKs/STAT3 signaling pathway. Moreover, IL-1 was shown to induce LIF expression and downstream JAK/STAT to generate iCAFs (130). On the other hand, TGF-β antagonizes the generation of iCAF by downregulating IL-1R expression, which promotes shifting to myCAFs. This phenotypic shift of CAFs results in a significant decrease in tumor volume (130). The EGFR is overexpressed in more than 90% of clinical patients. Many studies show that EGFR activates MAPK and JAKs-STAT3 signaling pathways, and STAT3 is involved in the survival of cancer stem cells (CSCs) (131). The JAK/STAT3 signaling pathway is vital in mediating the effects of IL-6 on tumor cell proliferation, survival, invasion, and metastasis. Reports show that selective targeting of STAT3 in cancer could provide multiple benefits, including inhibiting cell-autonomous effects on tumor cell growth and metastasis. Therefore, therapies targeting EGFR and IL-6 pathway components could be used to impair STAT3 activation and signaling (132). Therefore, manipulating the JAKs/STAT3 signaling pathway is a promising approach for metastatic CRC treatment.

Reports show that reduced STAT3 signaling results in the loss of stem cell maintenance, while STAT5 and STAT1 primarily affect cellular survival. Although higher levels of STAT5 were reported in hematological malignancies, recent findings show that STAT5 also mediates solid tumorigenesis (133). STAT5A knockout mice showed defects in progesterone signaling resulting in undeveloped mammary glands and pregnancy difficulties. However, STAT5B knockout mice display dwarfism, lower hepatic RNA biosynthesis capacity, reduced glucose and lipid metabolism, and sexual conversion with marked gender differences (11). It was shown that STAT5A and STAT5B knockout strains are viable. In addition, the Stat5 double knockout embryos are anemic, leukopenic, had smaller spleens and thymi, and disordered thymic architecture, which results in severe combined immunodeficiency phenotype (134).

Evidence indicates that the JAK2/STAT5 pathway is activated in different cancers, suggesting that the AK2/STAT5 signal could be a promising drug target. The biological processes related to JAK2/STAT5 pathway are mainly triggered by transcriptional activation of STAT5 target genes. Recently Wang et al. (135) studied the expression of Matrix Gla protein (MGP) protein in both gastric cancer (GC) and normal tissues. The results show an association between MGP and STAT5 signaling. In addition, the biochemical assays revealed that binding of MGP promotes phosphorylated-STAT5 (p-STAT5), which leads to the suppression of GC cell apoptosis through activating the transcription of downstream genes. In addition, the application of STAT5 inhibitors suppressed the oncogenic effects of MGP, suggesting that GC patients with high levels of MGP expression may show increased sensitivity to STAT5 inhibitor treatment (135). Moreover, there are variations in the association between p-STAT3 and survival in colon cancer, but a high p-STAT3/p-STAT5 ratio indicates a bad prognosis (136). Unphosphorylated STAT5A helps to stabilize the heterochromatin upon binding to heterochromatin protein 1α(HP1α) and acts as a tumor suppressor. The transcriptome profiling study showed that unphosphorylated STAT5A could repress several genes (e.g., *TGFB1* and *FOXQ1*) involved in colon cancer development (137).

The JAK/STAT signaling pathway also makes epigenetic changes that alter gene expression. For instance, histone acetyltransferase p300/CBP can execute acetylation on STAT3, which recruits DNA methyltransferase 1 (DNMT1). STAT3, STAT5A, and STAT5B are highly expressed in most cell types. It was demonstrated that deletion of STAT3 in mice results in embryonic lethality, while deletions of STAT5a and STAT5b lead to developmental and immune defects, respectively (138). Genome-wide analyses by Mandal et al. (139) revealed that STAT5 tetrameric binding motif is associated with transcriptional repression in leukemias. The tetrameric STAT5 is also shown to recruit Ezh2, repressing several genes regulated by STAT5 during B lymphopoiesis (139).

## The interaction between m6A and JAKs-STAT3/5 during CRC

**Figure 3:**
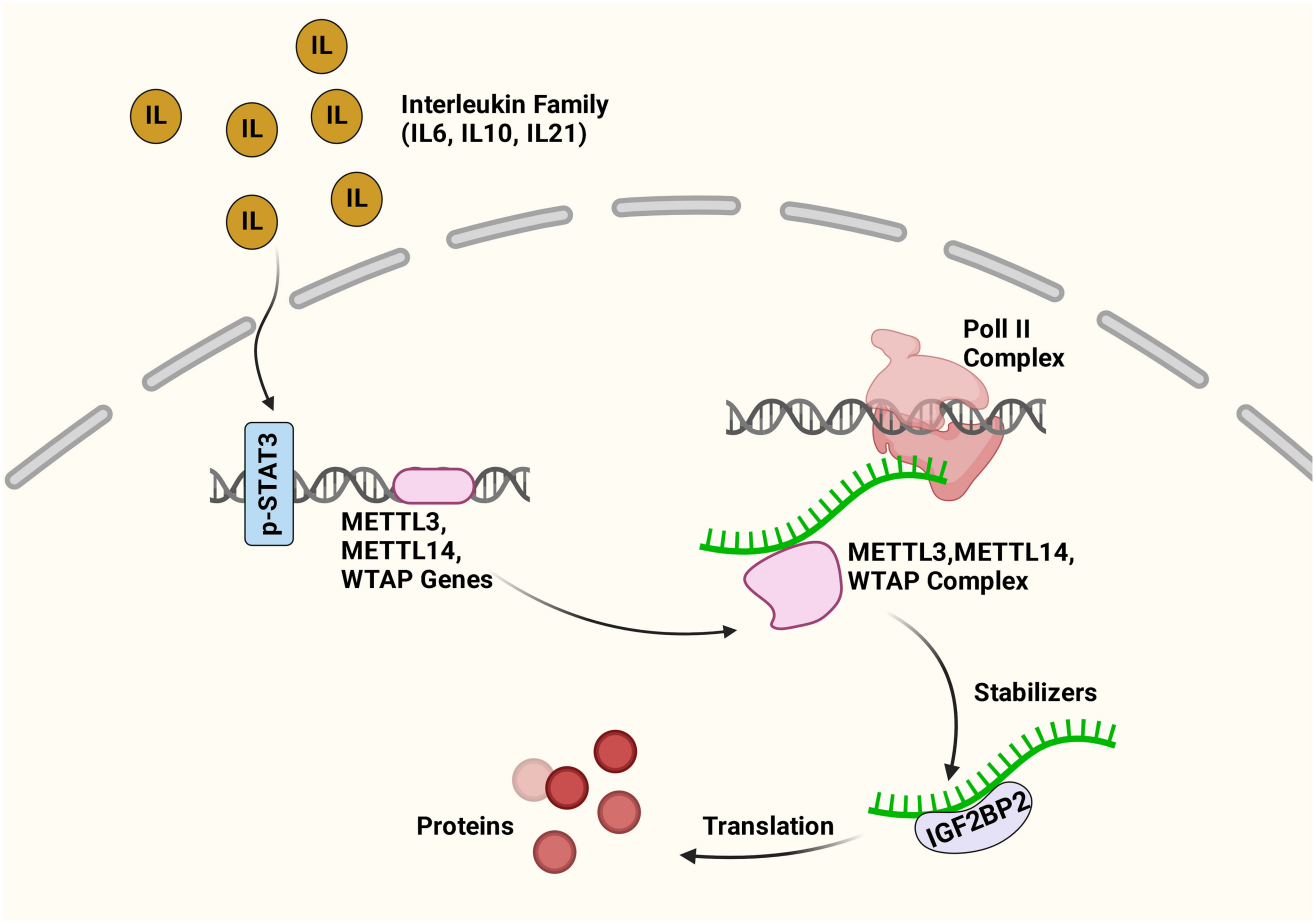
Cross-talk between m6A and STAT3 pathways. The inflammatory factors activate the STAT3 pathway. The activated STAT3 increases the expression of METTL3, METTL14, and WTAP genes, consequently enhancing the expression of m6A genes. IGF2BP2 can recognize the methylated mRNA transcripts, and the interaction maintains mRNA stability and expression. Figure created with BioRender.

Compelling evidence shows that m6A modification governs the expressions and functions of ncRNAs, thus controlling cancer stemness properties. On the other hand, the JAK/STAT3 signaling pathway is important in cancer stemness research as it can link ncRNA and m6A in tumorigenesis and metastasis (64). It was shown that the upregulation of lncRNA ITIH4-AS1 leads to downregulation or depletion of RE1 silencing transcription factor (REST) in CRC, which consequently promotes ITIH4-AS1 expression and induces tumor proliferation and metastasis through JAK/STAT3 pathway (140). Activated JAK1/STAT3 is crucial in gastric cancer proliferation and metastasis (141). Recent findings demonstrated that m6A modification regulates the key molecules in the JAK/STAT3 signaling pathway. The proposed interaction between STAT3 and m6A signaling pathways is presented in **Figure 3**.

Reports show that the SOCS family modulates several cytokine induced intracellular signal pathways. For instance, SOCS2 regulates different biological processes, such as immune responses (142, 143). SOCS2 is triggered by tyrosine phosphorylation and functions downstream of the JAK/STAT pathway, consequently negatively regulating this signaling pathway (144). It has been shown that the dysregulation of the JAK/STAT signaling pathway is very common in gastric cancer (145). Recently, Jiang et al. (146) used an AGS (human gastric cancer cell line) culture. They showed no difference between STAT1 and STAT3 regarding tyrosine phosphorylation in the MELLT3-KO AGS cells compared to wild-type cells. However, the results of this study demonstrated a negative association between SOCS2 and cell proliferation in gastric cancer cells. In addition, METTL3 knockdown enhanced SOCS2 expression, reducing cell proliferation in AGS cells (146). A study by Wu et al. (147) revealed that expression of JAK2 and SOCS3 due to the loss of METTL3 results in impaired self-renewal capacity and triggers the differentiation of induced pluripotent stem cells.

A study on IL-7/STAT5/SOCS pathways explored the involvement of RNA modifications in T-cell homeostasis (148). Li et al. (148) showed that the mRNAs of the suppressor of cytokine signaling (SOCS) gene family are labeled by m6A enzymes which result in higher mRNA levels in Mettl3-deficient immature T cells. In addition, higher activities of the SOCS gene family could also inhibit the activation of IL-7-mediated STAT5 and T-cell homeostatic proliferation and differentiation (148). Also, m6A modification through METTL3 seems essential for T regulatory cell (Treg)-suppressive functions via IL-2/STAT5 signaling (149), and Treg cells that lack METTL14 cannot inhibit colitis in mice (150). The m6A modification of Jak1 mRNA in tumor-infiltrating myeloid cells (TIM) via METTL3 improves the translation efficiency of JAK1 protein and STAT3 phosphorylation (151). It was shown that low expression of YTHDF2 in multiple myeloma reduces cell proliferation. The RIP sequencing study of m6A revealed STAT5A as a downstream target of YTHDF2, and its binding to the m6A modification site of STAT5A enhances mRNA degradation (152). The role of YTHDF2 in tumor progression has been well-studied. However, the beneficial function of YTHDF2 in the immune response to tumor cells has been recently discovered. YTHDF2 was shown to be upregulated in natural killer cells, a key component of innate immunity, upon activation by cytokines, tumors, and cytomegalovirus infection. YTHDF2 also maintains natural killer cell homeostasis and maturation by establishing a STAT5-YTHDF2 loop (153).

A recent study by Fang et al. (82) emphasized the role of ALKBH5 in gastric cancer. They showed that ALKBH5 is significantly expressed in gastric cancer samples enhancing gastric cell proliferation and metastasis. ALKBH5 also removes the m6A modification of JAK1 mRNA, leading to upregulation of JAK1 expression mediated by LINC00659 in an m6A-YTHDF2-dependent manner, consequently activating the JAK1/STAT3 pathway in gastric cancer. In addition, the ALKBH5 silencing disrupts gastric cancer tumorigenesis via the JAK1 axis (82). A recent study showed that ALKBH5 regulates the activity of STAT3 in osteosarcoma in an m6A-YTHDF2-dependent manner. It was demonstrated that YTHDF2 could read SOCS3, leading to higher levels of m6A-methylated transcript degradation (154). In addition, FTO could negatively regulate STAT3-mediated signaling and induce pro-inflammatory IFN-stimulated genes (ISGs) during the IFN response. Depletion of FTO led to higher phosphorylation and activation of transcription factor STAT3 (155). The upregulation of lncRNA ITIH4-AS1 in colorectal cancer enhances RE1 silencing transcription factor (REST) downregulation or depletion, which consequently upregulates ITIH4-AS1 and promotes tumor proliferation and metastasis through JAK/STAT3 pathway (140).

In general, STAT5 plays a crucial role in the development and differentiation of various cells in the body, including immune cells. In bowel disease, STAT5 is involved in the pathogenesis of IBD and colorectal cancer. STAT5 activation is decreased in patients with IBD, leading to impaired immune function and dysregulation of the intestinal epithelial barrier. This dysregulation of the epithelial barrier can lead to increased intestinal permeability and bacterial translocation, contributing to the pathogenesis of IBD. Additionally, STAT5 plays a role in regulating the differentiation of T cells, which are essential in the immune response in the gut. Dysregulation of STAT5 signaling is associated with an imbalance in T cell populations and the development of IBD.

On the other hand, m6A is a chemical modification of RNA that affects mRNA stability, splicing, and translation efficiency. Growing evidence suggests that there is cross-talk between STAT5 and m6A signaling pathways. For example, STAT5 directly interacts with the m6A methyltransferase METTL3, which adds m6A to mRNA. This interaction leads to increased m6A methylation of STAT5 target genes, resulting in their destabilization and reduced expression. In addition, m6A modification can regulate the activity of STAT5 by affecting the stability and translation efficiency of STAT5 mRNA. Increased expression of METTL3 in colorectal cancer demonstrated dual functionality in gene regulation, encompassing both methyltransferase activity-dependent and -independent functions. METTL3 promotes JAK1 translation by adding m6A modifications to the 3’ untranslated region of the JAK1 transcript.

**Figure 4:**
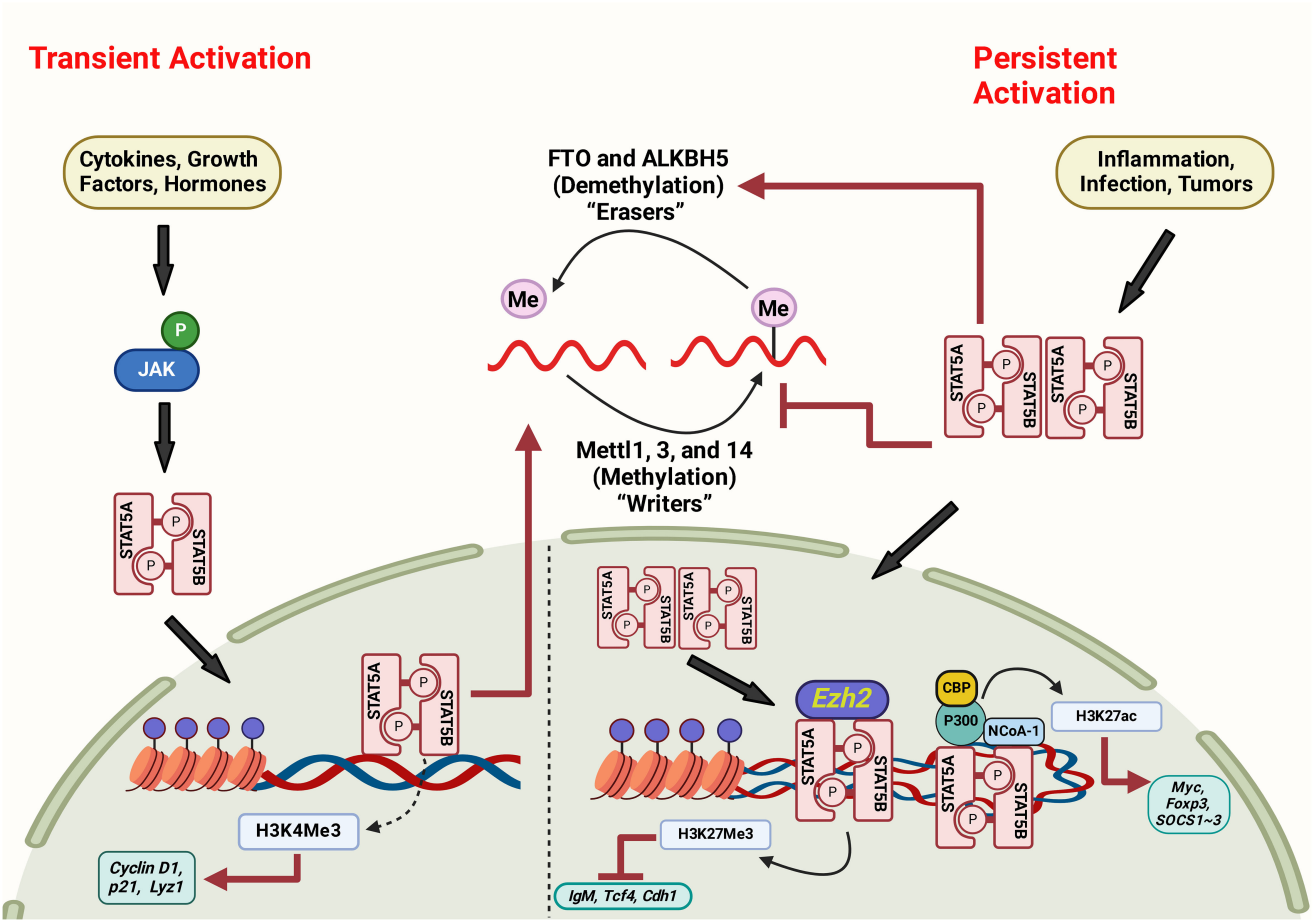
The interaction between STAT5 and m6A pathways via persistent and transient activation mechanisms. Figure created with BioRender.

Additionally, redistribution of METTL3 to the STAT3 promoter in collaboration with NF-κB enhances STAT3 transcription, independently of its methyltransferase activity. The concurrent elevation of JAK1 and STAT3 contributed synergistically to activating the p-STAT3 signaling pathway, subsequently increasing cancer cell proliferation and metastasis (156). **Figure 4** illustrates the possible interaction between STAT5 and m6A pathways (**Figure 4**). Overall, it is suggested that a complex interplay between STAT5 and m6A signaling pathways can have important implications for gene regulation and cellular function. Further research is needed to understand better the molecular mechanisms underlying this crosstalk and its relevance to human disease, including gastric cancer.

## Conclusion

CRC is a frequent tumor malignancy with high incidence and mortality worldwide. The epidemiological data indicate that the number of CRC patients is increasing annually in developing and developed countries, and the progress of CRC seriously threatens the survival of patients. Also, the advanced stage of CRC patients has poorer outcomes. Due to the lack of diagnostic biomarkers and therapeutic targets, CRC treatment is disappointing. Thus, further exploration of the underlying molecular mechanisms of CRC progression is inevitable (157). The N6-methyladenosine is the most common mRNA modification crucial in tumor metastasis in various cancers. Therefore, exploring how RNA m6A modification is regulated in CRC recurrence and metastasis is of great interest in improving CRC patient prognosis.

The intestinal epithelium undergoes continuous self-renewal to maintain its integrity and functionality. The role of m6A modification in regulating cell differentiation and proliferation in intestinal epithelial cells (IEC) is an important aspect of its function in maintaining gut homeostasis. The m6A modification can regulate the expression of genes that control the commitment of intestinal stem cells to specific lineages, such as absorptive enterocytes, mucus-secreting goblet cells, hormone-producing enteroendocrine cells, and antimicrobial peptide-producing Paneth cells. By affecting the stability and translation of lineage-specific transcripts, m6A modification helps direct stem cells toward different cell fates (158). The JAK/STAT pathway also contributes to the regulation of cell fate decisions in intestinal epithelial stem cells and progenitor cells. Activation of specific STAT proteins can drive gene expression in lineage specification. For instance, STAT3 activation has been linked to the differentiation of intestinal stem cells into absorptive enterocytes, while STAT6 activation may promote the differentiation of goblet cells that secrete mucus. Proper cell turnover in the intestinal epithelium requires balancing cell proliferation and cell death. m6A modification can impact gene expression in cell cycle progression, cell growth, and survival. The JAK/STAT pathway also influences the differentiation and proliferation of IECs. Properly balanced proliferation is essential for maintaining tissue integrity and preventing the accumulation of damaged cells (11). Dysregulation of m6A modification and/or JAK/STAT pathway can disrupt this balance, potentially leading to uncontrolled cell proliferation or impaired differentiation, which is associated with conditions like colorectal cancer (159). Conversely, inadequate activation of the pathway may impair regeneration and healing after injury. The JAK/STAT pathway and m6A modification also contribute to forming and maintaining an epithelial barrier that separates the body’s internal environment from the external environment within the gut lumen.

The m6A levels are determined by m6A writers (METTL3/METTL14/WTAP protein complex) m6A erasers (FTO and ALKBH5) and m6A readers (YTHDC1–2, YTHDF1–3, IGF2BP1–3, HNRNPC, and HNRNPA2B1) (1). The collaboration between writers, erasers, and readers of m6A methylation was shown to participate in the progression of various types of tumors. The m6A modification consequently facilitates the recruitment of m6A readers that link m6A-modified RNAs to mRNA processing enzymes, affecting RNA export, splicing, translation, and degradation (39).

The m6A-profiling methods require a large amount of RNA material; thus, m6A distribution profiling in the transcriptome of patient samples, particularly cancer stem cells and the primitive/progenitor cells of normal tissues will be challenging. The emergence of new techniques that utilize a small amount of RNA and provide base-resolution m6A profiles with better quantitative information is desired. Also, CRISPR genome editing and CRISPR-mediated RNA modification approaches would provide informative information about the epistatic relationship between RNA methylation and chromatin dynamics (15). The existence of cancer stem cells (CSCs) in colorectal cancer has been recently indicated. Their role in metastasis, drug resistance, and continual adaptation of cancer cells to the changing tumor microenvironment (TME) has also been discovered. In addition, the accumulation of epigenetic and genetic variability leads to the evolution of the CSC, consequently resulting in tumor growth and maintenance. Thus, exploring key genes involved in transforming tumor CSC and unraveling the underlying mechanisms in colorectal cancer may uncover novel therapeutic targets (160). Although there is mounting evidence of the involvement of m6A in CRC, the expression and functional effects of m6A RNA methylation on CRC are still poorly understood.

The JAK/STAT pathway was shown to be involved in abnormal gene expression related to high cytokine levels. Studies show that JAK/STAT inhibitors could be effectively used to treat multiple diseases, such as rheumatoid arthritis (RA) and systemic lupus erythematosus (SLE), indicating that JAK/STAT serves a vital role in disease development (161, 162). Chronic inflammation was shown to drive tumorigenesis. The JAK/STAT signaling pathway can link inflammation with cancer. JAK/STAT is one of the leading 12 signaling pathways abnormally regulated in cancer. The function of STAT5 in intestinal homeostasis has been proven. It was shown that STAT5 plays an essential role in the interaction regulation of microbiota and IEC, mediating chronic inflammation and promoting mucosal healing. The upregulation of JAK/STAT signaling was shown to be involved in cancer aggressiveness and tumor progression. The increased JAK/STAT signaling in different cancer diseases, including CRC, impairs prognosis and decreases overall survival.

Since JAK/STAT signaling pathway upregulates different aspects of cancer development, including cell growth, differentiation, and survival, its inhibition could be employed as a potential strategy for cancer. Decreased m6A methylation in cells reduces the STAT3 activity, leading to lower cell proliferation, while up-regulation of STAT3 could reverse its effects on cell growth (154). Recently, Fang et al. (82) reported the phosphorylation of STAT3, but not STAT1 or STAT5, by JAK1 in gastric cancer cells. The JAK1 upregulation promoted the STAT3 phosphorylation and activated the JAK1/STAT3 pathway, which accelerated the progression of gastric cancer. M6A is involved in the expression of different transcripts of the STAT3 signaling pathway resulting in activation or inhibition of the STAT3 signaling pathway in various tumors (154). In cholangiocarcinoma tumor cells, the binding of STAT3 to the m6A writer gene results in the upregulation of m6A writers by cytokine IL-6, suggesting that m6A is a potential target in response to inflammation (163). In cancer stemness research, the JAK/STAT3 signaling pathway is pivotal in linking ncRNA and m6A in tumorigenesis and metastasis (64).

In conclusion, the findings emphasize the vital function of m6A RNA methylation and the JAK/STAT signaling pathway in developing various diseases. Thus, deciphering the underlying molecular mechanism and the interplay of these two mechanisms will help us better understand the development of human diseases and provide us with more sophisticated tools to treat diseases in the future. Studies indicate that m6A modification regulates the key molecules in the JAK/STAT3 signaling pathway. Loss of METTL3 affects JAK2 and SOCS3 expression patterns, leading to impaired self-renewal capacity and triggering the differentiation of induced pluripotent stem cells (164). Generally, loss of m6A modification leads to slow mRNA decay while it increases expression of the STAT signaling inhibitory proteins SOCS1, SOCS2, and CISH, consequently inhibiting cytokine-mediated STAT5 activation, T cell proliferation, and T cell differentiation (165).

## The perspective of the role of m6A in the JAKs-STAT3/5-induced GI cancer

Studying other m6A-related regulatory factors is necessary, particularly in CRC. It has been proven that the poor prognosis of CRC has been strongly linked to the abnormal expression of m6A regulatory factors. While numerous cellular signaling pathways have been established as contributors to CRC metastasis and their underlying molecular mechanisms have been extensively explored, the complete understanding of their interaction and regulation in CRC progression remains elusive. STAT5 regulates microbiota interaction, and IEC mediates chronic inflammation and promotes mucosal healing. However, the function of STAT5A and B in promoting healing during IBD disease and cancer is not fully understood. Therefore, there is an urgent need to explore the underlying mechanism of the JAK/STAT pathway in intestinal homeostasis to illustrate its involvement in colorectal cancer formation. In addition, more experimental studies are required to attain solid evidence to identify the crosstalk between JAK/STAT and m6A methylation as a possible prognostic biomarker of inflammation and infection and therapeutic developments.

## Author contributions

NE: Conceptualization, Validation, Writing – original draft. AB: Writing – original draft. WT: Supervision, Writing – review & editing. SL: Supervision, Writing – review & editing. XH: Conceptualization, Funding acquisition, Supervision, Writing – review & editing, Validation.
